# Student nurses’ views on shift patterns: What do they prefer and why? Results from a Tweetchat

**DOI:** 10.1002/nop2.1208

**Published:** 2022-03-21

**Authors:** Chiara Dall’Ora, Jessica Sainsbury, Chris Allen

**Affiliations:** ^1^ NIHR ARC Wessex Wessex UK; ^2^ School of Health Sciences University Of Southampton Southampton UK; ^3^ Solent NHS Trust, and seconded at the Florence Nightingale Foundation London UK

**Keywords:** 12‐hr shifts, shift work, student nurses, thematic analysis, Twitter

## Abstract

**Aim:**

The main aim of the study was to understand student nurses’ views around shift patterns.

**Design:**

Qualitative study.

**Method:**

We held a Tweetchat in May 2019, where we asked questions around the frequency of 12‐hr shifts working on placement; schedule flexibility while on placement; which shift patterns they preferred and why. Data from the Tweetchat were analysed using reflexive thematic analysis to generate themes from initial codes.

**Results:**

Seventy‐three nursing students participated in the Tweetchat. The majority reported that they work 12‐hr shifts on placements, particularly when based in a hospital. We identified three themes: ‘Achieving a personal equilibrium’; ‘Meeting the needs of the care environment’; ‘Factors affecting negotiation capacity’. Data highlighted a conflict for most students, where they preferred 12‐hr shifts because of more time off for study, paid work and leisure, while acknowledging 12‐hr shifts negatively affected their fatigue, exhaustion and led them to follow a poor diet and neglect exercise and sleep.

## 1 INTRODUCTION

The debate on whether nurses should work 12‐hr shifts has been ongoing for more than 40 years (Dall'Ora & Dahlgren, 2020). Large multi‐country studies of thousands of nurses across Europe and the US have shown negative impacts of 12‐hr shifts on both patient care and on outcomes of nurse wellbeing and performance (Clendon & Gibbons, 2015; Dall'Ora et al., 2015; Dall'Ora, Griffiths, et al., 2019; Griffiths et al., 2014). Nonetheless, anecdotal reports that many nurses prefer working long shifts are frequent, with better work‐life balance and costs often cited as main motivators (Ejebu et al., 2021).

## 2 BACKGROUND

Despite growing qualitative evidence around nurses’ views of 12‐hr shifts, research on student nurses' perceptions and opinions of shift patterns is currently missing. In the UK, student nurses tend to exhibit different characteristics from staff nurses, especially students who have enrolled since 2018, who receive less financial support. Student nurses face multiple challenges, including combining academic study and placement demands. In particular, students with families and caring responsibilities are confronted with competing demands in managing and balancing their student and family life (O'Brien et al., 2017). It is not uncommon for student nurses to seek part‐time employment, with reports of up to 35 hr per week (Rochford et al., 2009) to support their living expenses and studies (Hasson et al., 2013).

The extent to which any of these factors influence student nurses’ preferences and views on shift patterns is unknown. Furthermore, it is unclear as to the extent to which student nurses are involved in long shift patterns, and the amount of flexibility they have in negotiating their shifts. There are anecdotal reports of students being able to negotiate their rotas on placement (Elliott, 2020; Rawson, 2020), but the spread of this practice across the UK is currently unknown. This study aims to explore which shift patterns student nurses in the UK work, which factors are important for student nurses to design their schedule, where allowed, and what their views and preferences of different shift patterns are.

## 3 DESIGN

This was a qualitative study of student nurses’ views on shift patterns, obtained through a TweetChat and analysed using reflexive thematic analysis (Braun & Clarke, 2021).

## 4 METHOD

We held a Tweetchat on 2 May 2019, hosted by *The Student Nurse Project* Twitter account. The Student Nurse Project is an online community where student nurses discuss current nursing issues.

Twitter is increasingly used in healthcare research, not only as a means of dissemination of studies’ findings, but also to collect data on a public platform, where it offers the opportunity to gather a large amount of data in a fast and cost‐free manner (Sinnenberg et al., 2017). The views of student nurses have also previously been gathered successfully for research, using such an approach (Clyne et al., 2018).

The Tweetchat occurred over a unique session for an hour and was advertised by The Student Nurse Project for a week before the Tweetchat. A pre‐chat blog was written, where all potential participants were informed about how the Tweetchat would work and specified that data obtained would be used for research purposes. Whilst at the time of the Tweetchat the Student Nurse Project account had 7,500 followers, the exact number of potential participants is unknown as many of the followers are Registered Nurses and academics, which were not the focus of our study.

The questions, which were sent 12 min apart, can be seen in Table 1. Participants were able to respond to questions for a further month, at which point the data were extracted. This also allowed participants to delete their responses, if they later decided they did not want to be included in the research.

**TABLE 1:** Questions asked during Tweetchat

Question Number	Question
1	As a student nurse, how often do you work 12‐hr shifts on placement?
2	As a student nurse, do you have a say in your rota while on placement? To what extent do you have a say?
3	As a student nurse, what shifts and shift patterns do you prefer and why?
4	As a student nurse, focusing on 12‐hr shifts, what do you like about this type of shift?
5	As a student nurse, remaining focused on 12‐hr shifts, what do you dislike about this type of shift?

## 5 ANALYSIS

Data from the Tweetchat were analysed using reflexive thematic analysis (Braun & Clarke, 2021) to generate themes from initial codes, as part of an active and interpretive process. All authors were involved in the process of coding the data and the themes were generated through ongoing reflexive discussion. The authors of this paper consist of an academic working in workforce research, a nurse academic working in education and a student nurse (at the time of data collection); therefore, the authors have varying perspectives and pre‐existing knowledge around the phenomenon of shift patterns in nursing students. This allowed us to balance our knowledge with accounts from student participants. We did not develop any a‐priori themes. To ensure trustworthiness and rigour we followed the step‐by‐Step approach for conducting a trustworthy thematic analysis as outlined by Nowell and colleagues (Nowell et al., 2017). This included prolonged engagement with data; reflexive journaling; peer debriefing; researcher triangulation when searching for themes; themes and subthemes vetted by all researchers at all stages; and keeping an audit trail. We also extracted quantitative data, including the proportion of student nurses working long shifts, the proportion of students who can self‐schedule and which shift patterns they prefer.

## 6 ETHICS

We obtained ethics approval for this study from the University of Southampton Ethics Committee (Approval ID: 48131). Participants’ confidentiality could not be maintained during the Tweetchat, as Twitter is a publicly available platform that anyone can access. Participants were aware that their tweets would be used for research and therefore, whilst consent was not formally taken, it was implied through their participation. We assigned a pseudoID to all participants (pseudoIDs start with ‘STN’ = ‘Student Nurse’) and did not retain any personal data of participants when extracting, analysing, and reporting data.

## 7 RESULTS

Seventy‐three nursing students took part in the Tweetchat and generated 252 Tweet interactions. Two interactions were off‐topic, and we did not include them in our analysis. Sixty‐eight per cent of respondents reported that they mostly work 12‐hr shifts on placements, while 27% identify a mixture of long and short shifts, often depending on the type of placement (e.g. longer shifts in acute ward placements as opposed to short shifts in community placements). Only two students reported working mostly 8‐hr shifts on placement. For data on the degree of flexibility and ability to influence their placement rota and shift patterns preferences, please see Table 2.

**TABLE 2:** Student nurses report of typical shift patterns, degree of say when planning rotas and shift preferences

Which shift patterns do you normally work?			As a student nurse, do you have a say in your rota?			Which shift patterns do you prefer?		
Mostly 12‐hr	Mostly 8‐hr	Mixture	Yes/mostly yes	No/mostly no	Sometimes	8‐hr	12‐hr	Depends/uncertain
25 (68%)	2 (5%)	10 (27%)	18 (58%)	3 (10%)	10 (32%)	5 (18%)	17 (61%)	6 (21%)

We identified three themes when looking at students’ narratives: achieving a personal equilibrium; meeting the needs of the care environment; factors affecting negotiation capacity.

### 7.1 Achieving a personal equilibrium

When expressing their preference for 8‐ or 12‐hr shifts, it became apparent that for students achieving a personal equilibrium was of vital importance. The need for personal equilibrium was expressed in terms of financial, learning, personal network work and physical and mental health needs, which we identified as subthemes. 12‐hr shifts both helped and hindered the attainment of these.

#### 7.1.1 ‘Most of us also have paid work’: Financial needs

A large number of students agreed that when finances were considered, 12‐hr shifts were preferable in terms of reduced travel costs (for example, fuel and parking to cover 3–4 days per week) and increased capacity to do paid work (working longer shifts gives more ‘whole days off’):

(I prefer) long days, 3 days a week‐ gives me time to get a shift in for £. And cost is massive for me‐ I struggled last year doing 4/5 days a week financially for petrol/parking. (STN9)

Some students mentioned that besides travel time and ability to engage in more extra paid work, finding parking was also an issue on 8‐hr shifts, especially when these were later in the day. Childcare costs were also relevant for some students.

#### 7.1.2 ‘Juggling deadlines and group work’: Learning needs

When considering learning needs, especially practice learning, students appeared to prefer 12‐hr shifts, presumably due to the longer consecutive time they offered on a shift, where they could engage in less fragmented activities, compared with 8‐hr shifts:

I think (12‐hour shift working) it has taught me the routine of the ward well, I have also been able to form relationships with patients as you can spend more time with them. (STN43)

Due to having more days off with 12‐hr shifts, students reported preferring them in terms of having more time to complete university work and catch up on missed placement hours. It was also apparent that students working shorter shifts occasionally ended up doing many of these in a row, which they noted was ‘counterproductive to learning’ (STN44).

For some students, however, working 12‐hr shifts was so tiring that university work felt compromised:

If I do three (12‐hr shifts) in a row the tiredness can then affect my ability to do uni work. (STN13)

The participants’ views on 12‐hr shifts, reflect some of the challenges experienced by students enrolled on healthcare programmes, with regard to balancing the practice related requirements of the course, alongside the programme's academic requirements. Currently in the UK, students need to complete at least 2300 practice hours across the length of their programme. This is complemented with a further 2300 theory hours (Henriksen et al., 2020). Because of these demands, it is common for nursing degrees to run into university holiday periods, where students on other degrees have opportunities to explore extracurricular activities.

#### 7.1.3 ‘I don't see the kids for days’: Personal network work

Having time to engage in personal networks, including family, caring, social and ‘life admin’ activities was a relevant factor in students’ apparent preference for 12‐hr shifts.

However, some acknowledged that traditional childcare arrangements were not ideal when working 12‐hr shifts:

I prefer longer days, allowing me more time off to spend with my kids, (although finding childcare for shift working is difficult). (STN21)

For some, working long shifts, meant ‘not see[ing] [their] kids for days’ and not being able to engage in activities with them during the day:

Mother guilt, I feel guilty leaving before (my children) wake, guilty that it's nearly their bedtime when I get home and being tired on my days off. (STN21)

It was common for students to face role conflict. This was most visible when students discussed shifts preventing them from doing the school run and other activities that were aligned to the role of parent. Whilst such conflicts have been noted in the context of nursing elsewhere (Zandian et al., 2020), there is currently limited recognition of such conflicts in pre‐registration nursing programmes. Such conflicts often resulted in a preference towards longer shifts. However, when considering activities such as housework and personal appointments, students indicated a preference for 8‐hr shifts:

I prefer 8 hours. Yes, it takes up 5 days of my week but it's a week pattern I'm used to. If I'm on earlies, it means I finish at 3.15 and still have time to do other things like book appointments after 3. (STN18)

#### 7.1.4 ‘It's a recipe for exhaustion, fatigue and ultimately burnout’: Physical and mental health

Whilst many student nurses reported preferring 12‐hr shifts due to the reduced travel costs and having more days off (often to allow them to work elsewhere), they mostly acknowledged that working 12‐hr shifts (especially where these were used to allow for remunerated employment) paid quite a severe toll on their physical and mental health:

I dislike 12‐hour nights. I'm so exhausted a week after. On days: you still likely get 1 break which you would on an 8 and so you're hungry and can't think straight which leads to mistakes. 12 hours + commute is a long time, not enough time to sleep. (STN46)

The literature links both shift work, and long working hours, (both commonly experienced by the included students) with negative outcomes, including burnout and fatigue, the genesis of long‐term illness, obesity and reduced job performance (Caruso, 2014; Dall’Ora et al., 2015). Besides fatigue and sleeping patterns students voiced their concerns about how long shifts have an impact on their ability to follow a healthy diet and get enough physical exercise:

I really struggle with eating on long days, I always plan to take a meal and snacks. But I end up eating 4 meals or just a pile of rubbish. I have gained nearly a stone and a half since I started University! (STN43)

I also find it harder to schedule in regular and consistent exercise week‐on‐week. (STN22)

Such concerns led to some students having a preference for shorter shifts, when they were able to do these. These concerns are echoed elsewhere in the literature and it is known that shift work, more generally, is associated with negative health outcomes, including metabolic syndrome and obesity (Zhang et al., 2020).

Impact on mental health was recognized by many, who recognized how working long shifts and, in addition, booking in extra paid work, and assignments, could lead to feelings of exhaustion and eventually burnout:

12.5 shifts also not healthy as a student nurse when most of us also have paid work. I have worked 60+ hrs when on placement. It's a recipe for exhaustion, fatigue and ultimately burnout. No time for meals or sleep. (STN45)

Such trends reflected the large number of unpaid placement hours, the cost of attending university, and the need to engage in supplementary paid employment to meet students’ financial needs and continue the programme. The financial challenges associated with undergraduate nursing programmes are noted across the literature (He et al., 2018; Van Hoek et al., 2019) and were very visible in the student's narratives.

### 7.2 Meeting the needs of the care environment

When students responded to questions about pros and cons of 12‐hr shifts, they also reflected on how these shift patterns promote or hinder meeting the needs of the care environment, in terms of continuity of patient care and workload demands, which we identified as subthemes.

#### 7.2.1 ‘More time with my patients and continuity of care for them’: Care continuity and holistic care

As seen in the literature with Registered Nurses (Baillie & Thomas, 2019), several students displayed a preference for 12‐hr shifts in terms of continuity of care. This was said mostly around the concept of ‘getting to know the patients better’, completing more care and avoiding miscommunication of information due to fewer handovers:

Personally, I prefer 12.5 hour shifts as it gives me a chance to get to know my patients for the day and follow their treatment throughout the day to the full extent, and it saves multiple transfers of care which could cause miscommunication. (STN26)

Some students thought that 12‐hr shifts also helped to give holistic care to patients.

I like how I can spend more time with service users, understand and empathise with them more which ensures I can provide holistic, person centred care as I know their goals, values etc. (STN3)

A few students raised the objection that while care continuity during the day might be increased, the higher number of days off resulting from 12‐hr shifts meant that students did not see patients for four days, which impacted on continuity of care and safety:

It also means 4 days I'm not seeing people in my care and less stability for them. (STN57)

#### 7.2.2 ‘It's not the staff's fault it's busy!’: Workload

Students were conscious that when it came to the impact of different shift patterns, high workload and lack of breaks were a determining factor for disliking 12‐hr shifts:

It can be physically exhausting working for 12.5 hours straight, especially on busy understaffed shifts when there's not much time for a break. Although supernumerary, I still feel the need to help out as if a substantive sometimes. (STN45)

However, some student nurses said that given the high pressures of their placements, they were compelled to help as much as possible, both because of concerns for how the team saw them, and concerns for their colleagues in practice and concerns for patients. One student said pressured to work long shift patterns because that was the expectancy of qualified nurses:

It can be hard to be supernumerary when you are still being judged for how well you behave as a nurse on a ward. There is so much pressure to get things done, and keep up with every other member of staff on the ward. I’ve had nurses tell me: you have to do 12 hours when you qualify. (STN63)

### 7.3 Factors affecting negotiation capacity

Our question around ability to choose and negotiate shift patterns elicited a variety of responses in student nurses’ ranging from some having no flexibility to complete ability to design their rotas gave NMC requirements for total hours were met. This is illustrated by the identified subthemes of placement lottery, personal circumstances, and fixed requirements.

#### 7.3.1 ‘I’ve been lucky so far’: Placement lottery

Students’ responses highlighted how variation in capacity to negotiate their shift patterns depends on the placement. This raises issues of equity between nursing students as it highlights the role of chance in determining how probably a student is able to negotiate shifts to accommodate meeting personal and occupational needs. Informal more flexible arrangements were available to some students, who were able to negotiate shifts around part‐time work and other commitments; though this was not a universal experience, raising issues of equitability in this aspect of many nursing programmes. Some students described how different placements had different stances about shift flexibility:

Depends on the placements, some get quite annoyed if you want to change only one day whereas the one I'm on now lets you change them around whenever as they say its our responsibility to get the hours done we need and to make sure we work with our mentors. (STN6)

In theory we work the shifts given to us, but in my own experience all my shift‐based placements have been very accommodating if I wanted to switch a few shifts around. (STN22)

These data were collected when students were required to spend at least 40% of their practice hours, working alongside their mentor, which influenced the shifts the students were able to do:

I've had to work when my mentor is working but normally this has been fine and I can pick which shifts of theirs suit me. (STN3)

Whilst this still allowed some negotiation, particularly if the mentor worked full time, those whose mentors were part‐time, or where there were many students placed in the same setting, invariably ended up having to follow the unsocial working patterns of their mentors:

I've been able to have quite a lot of flexibility where I've had full time mentors, where they've been part time I've had to basically do every shift they're on so I've ended up with tonnes of nights. (STN46)

Whilst this requirement limited some negotiation capacity, participants often prioritized working alongside their mentor as much as possible to get the best learning opportunities. This tended to lead to students working 12‐hr shifts, especially in acute environments where such shifts are now normalized (Dall'Ora et al., 2019). Indeed, 12‐hr shifts now appear to be something of a hidden curriculum (Raso et al., 2019) in many practice settings.

In addition, a few students described how asking for changes in shift patterns had to be approached with careful and considered diplomacy:

Our rotas are made for us and most wards will let you change a few shifts if need be‐ providing you go about it the right way and have a reasonable excuse for changing. (STN42)

You just have to approach them in the right way and remember flexibility works two ways. (STN12)

#### 7.3.2 ‘Caring responsibilities that sometimes require flexibility’: Personal commitments

In some cases, having personal commitments could be used as leverage to change shift patterns:

I've always had a say in my rota whilst on placement‐ however this may be due to me having caring responsibilities that sometimes require flexibility. I've never had a negative experience when asking to change shifts or when I can only work certain days. (STN24)

However, such a strategy was not always successful and reflected a tension between the requirements of the course and the need to maintain other roles:

I've never had a placement take my kids into account with off duty. I have missed plays and concerts. And always be told to get used to it. (STN62)

There was also some evidence that some providers took into account students’ needs to work part time:

On my ward placement I was able to take weekends off due to the fact I worked. I was always more than happy to work them if I didn’t have work but I was told that ‘your paid work lets you live make up your hours during the week so you can work’ which I appreciated. (STN2)

Overall, it appears that the most determining aspect of students’ flexibility is the ward/clinical setting where students are in placement and the normalized shift pattern of that environment. However, other factors may impact on students’ ability to negotiate their shift patterns.

#### 7.3.3 ‘All my ward based placements have been 12 hr shifts’: Fixed requirements

We further explored which aspects were detrimental to student ability to negotiate shift patterns. Students acknowledged how having placements in settings with fixed service hours was a limiting factor:

Monday to Friday placements such as out patients there's no negotiation. (STN44)

Some placements did not allow students to work long shifts, hence students experienced no negotiation capacity:

12 hours on a ward if I can, but I've had one ward that didn't let students do 12‐hour days and one that didn’t do 12‐hour days because although it was classed as a ward it was research so did short days. (STN23)

Compared with employed colleagues, student nurses have less say in where they work, and how they work. Registered Nurses, acting as employees, may be better able to negotiate specific patterns of work, that consider personal needs (i.e. childcare and caring responsibilities) and/or financial reasons (such as enhanced antisocial pay).

## 8 DISCUSSION

This study was the first to elicit student nurses’ views and experiences around shift patterns on placement. By analysing thematically 252 tweets by 73 student nurses, we showed that the majority work and prefer long shifts, and most can influence their rota on placement – though this is not consistently experienced, raising issues of equity. When exploring what students like and dislike about 12‐hr shifts, two main priorities were uncovered – namely *achieving a personal equilibrium* and *meeting the needs of the care environment*.

When it came to work‐life balance, students appeared to prefer long shifts, as these were offering more days off which could be used for engaging in social activities, caring for children and dependants and save money on parking and travel. Our findings mirror previous qualitative evidence from registered nurses and nursing assistants, where one of the reasons for preferring 12‐hr shifts was the extra days off they offer (Baillie & Thomas, 2019; Haller et al., 2018).

Notwithstanding the preference for long shifts, several students reported feeling ‘exhausted’ and ‘drained’ when working 12‐hr shifts. Fatigue is more common among nursing staff who work long shifts (Barker & Nussbaum, 2011; Thomson et al., 2017), indicating that the potential of long shifts to lead to negative well‐being outcomes for student nurses is high. Recovery after 12‐hr shifts is challenging, with nurses describing the first few days off as merely recuperation time, where they strive to make up for the lost sleep and the cumulative fatigue (Gifkins et al., 2020; Linsey M. Steege et al., 2014). However, a factor that makes working long shifts possibly more demanding for students is that they need to also complete academic work, including studying and writing assignments, and for many students, catch up on missed hours and complete additional paid shifts. This is of potential concern, especially considering that some students in the Tweetchat reported working more than 60 hours a week, which is considered to be an excessively long working week (Harma et al., 2015) and enhances the risk of lack of recovery from long shifts, which is essential to avoid the development of long‐term fatigue (L. M. Steege et al., 2018). Such excessive hours breach the European working time directive (Directive, 2003/88/EC, 2003), but are often necessary for students to be able to complete their programmes.

Despite expressing feelings of exhaustion, most students did not seem to believe the quality of care they provided was affected when working long shifts; it appeared that student nurses were aware of the fatigue *after* the shift, but only a few acknowledged the *within* shift fatigue caused by 12‐hr shifts. This neglect of in‐shift fatigue resembles that outlined by the ‘*Supernurse culture*’ construct, whereby nurses report experiencing fatigue, but believe it is a standard component of nursing; therefore, nurses feel they have the tools and skills to operate despite being fatigued, similar to superheroes having special powers (L. M. Steege & Rainbow, 2017). Moreover, the ‘*Supernurse’ culture* was also highlighted when students mentioned feeling compelled to help the understaffed and under resourced wards. While the desire to relieve pressure from strained health services is commendable, it appeared to motivate some students to prioritize the placements’ needs over their fundamental and learning needs.

Far from identifying 12‐hr shifts as a potential predictor for poor quality of care, most students said that working long shifts increased continuity of care and holistic care, since they would be seeing the same patients for longer on the same day. Some students perceived that having only one handover instead of two, across the 24 hr of the day led to a lower risk of miscommunication between shifts. Whilst these perceptions reflect how participants came to see such shifts, quantitative research of >30,000 nurses across 12 countries showed that 12‐hr shifts were not associated with increased continuity of care, and that no important information was missed at handover when shorter shifts were worked (Dall'Ora et al., 2020). In addition, a few students reported that having more days off could also lead to reduced continuity of care, highlighting that other factors may be more important in maintaining continuity of care (Sparbel & Anderson, 2000) than shift patterns.

When focusing on capacity to negotiate shift patterns, most students reported being able to influence their schedule; among the factors which might have increased capacity to negotiate there were personal commitments including childcare and part time work, although these were not always perceived by placements as valid reasons to have flexible shift patterns, hence there was always an element of chance.

Students’ accounts elicited the existence of a ‘*placement lottery’*, according to which a student may get completely opposite stances about work hours depending on where they are on placement. While this lottery construct does not apply to placements with fixed service hours, where choice of shift patterns is by definition constrained, it was seen in students’ recounts of feeling powerless in some placements and being involved in designing their rotas in other placements. Higher work time control can be beneficial for nurses, in terms of their sickness absence (Turunen et al., 2020) and wellbeing (Nijp et al., 2012), although any positive effects appear to be negated when healthcare professionals opt to work long shifts (Karhula et al., 2020). Nonetheless, there seems to be inequity in the degree of choice that students' nurses are given when it comes to shift patterns. Given the importance of student experience in determining the quality of university degrees (Lenton, 2015), providing more schedule flexibility may increase applications to nursing and open up nursing as a career choice to those with caring responsibilities. This is probably to be challenging in the context of increasingly constrained placement capacity and the many challenges placement areas are facing in the UK.

### 8.1 Limitations

There are several limitations to this study. Compared with in‐depth interviews, deploying a Tweetchat does not allow substantial breadth, depth or richness of data. Tweets are limited to 280 characters and therefore responses were typically brief. Whilst frequency is not a focus of qualitative research, a few of the themes were supported by only a few participants’ experiences. There was limited opportunity to further explore participant responses in the same way that could be achieved in semi‐structured interviews. Nonetheless, this study was the first to elicit student nurses’ views on shift patterns and thus can serve as a foundational point for future studies seeking to clarify the lived experiences, needs and preferences of student nurses. We did not collect any demographic or personal data. Future research should aim to collect such information to ensure the sample is representative of different universities and sociodemographic characteristics in the UK. In particular considering part time working patterns by socio‐demographic characteristics could help better understand who is more reliant on this extra paid work to support their studies.

Whilst it was intended that all participants were currently student nurses, this could not be guaranteed due participants being self‐selected. Where it was obvious that the tweet(s) came from someone who was not a student nurse, their data were not included. Because we did not collect personal data, full member checking was not possible. However, one of the authors of this paper was a student nurse at the time of data collection and analysis, and they gave constant feedback as to the creation of the themes and subthemes.

## 9 CONCLUSIONS

Whilst students acknowledge that the toll of long shifts on their physical and mental health is high, they seem to prioritize the extra days off over their well‐being. This highlights the increased pressures students are subject to, whereby they do not only need to achieve a work‐life balance, but a work‐university‐life balance, sometimes whilst being in practice for more than 60 hr a week. The little awareness of the impact of fatigue on well‐being, performance and patient safety is worrying, and should be addressed in nursing programmes when placements are planned. The inequality of students’ choice and negotiation around shift patterns should also be addressed by nursing programmes, who might act as advocates of equal student choice. Balancing this with placement capacity and availability is probably to be an enduring issue. It is the authors’ view that, given the high risk of harm that fatigued students might expose themselves and their patients to, any efforts both at a national and university level to financially aid students should be encouraged and pursued.

## CONFLICT OF INTEREST

The authors declare no conflict of interests.

## AUTHORS CONTRIBUTION

Chiara Dall’Ora: Conceptualization, Methodology, Formal Analysis, Writing – Original Draft Preparation and Writing – Review and Editing. Jessica Sainsbury: Methodology, Data Formal Analysis and Writing – Review and Editing. Chris Allen: Conceptualization, Methodology, Data curation, Formal Analysis, Writing‐ Original Draft Preparation and Writing – Review and Editing.

## Data Availability

The data for this study are not publicly available. Dr Dall’Ora and Dr Chris Allen confirm that they had full access to all the data in the study, and take responsibility for the integrity of the data and the accuracy of the data analysis.
